# From sensors to solutions: Integrating wearable sensor data into health research

**DOI:** 10.1002/alz70863_110570

**Published:** 2025-12-23

**Authors:** Kit B. Beyer, William E. McIlroy

**Affiliations:** ^1^ University of Waterloo, Waterloo, ON Canada

## Abstract

Integrating wearable sensor technology into research and clinical applications affords the opportunity to support aging individuals, including those living with Alzheimer's disease‐related dementias (ADRD), by advancing our understanding of disease onset and progression and enhancing personalized healthcare. Wearable sensors enable continuous remote capture of behaviour and physiology for prolonged durations as individuals participate in their daily lives. Objective, quantitative analysis of these real‐world data can yield important health‐related outcomes across multiple domains, including mobility, cognition, cardiovascular function, sleep, and physical activity. These outcomes can reduce reliance on subjective self‐reporting, improve the possibility of capturing infrequent events, detect subtle change over time, and provide a more comprehensive representation of an individual's health status.

However, the volume and complexity of wearable data also present several challenges and considerations that must be addressed to fully realize this opportunity. Data quality is critical to the validity and utility of wearable‐derived outcomes but is susceptible to many factors, including sensor calibration, device performance, participant adherence to wear protocols, and signal quality issues related to sensor malfunction, artifact, or noise. Complex, multi‐domain analysis that requires integrating data from multiple sensor types across different wearable devices is complicated by the many different data types and formats (e.g., standard vs. proprietary) used by various device manufacturers and the lack of built‐in capabilities to synchronize data across devices. Finally, advanced analytic techniques are often required to extract relevant features or events from large, complex wearable datasets.

This presentation will describe various approaches to address these challenges in processing and analyzing wearable data and demonstrate the opportunities that arise. Specific emphasis will be placed on 1) study design and implementation considerations that can mitigate challenges, 2) preprocessing, signal processing, and data analytics techniques that directly address these challenges, and 3) examples of the types of outcomes that can be derived from wearable data when these challenges are adequately addressed.

